# Wissenschaft und Praxis im Öffentlichen Gesundheitsdienst: Erfahrungen aus Sachsen-Anhalt und Perspektiven für einen zukunftsfähigen ÖGD

**DOI:** 10.1007/s00103-026-04242-6

**Published:** 2026-04-30

**Authors:** Doreen Wolff, Christian Apfelbacher, Robert Pohl, Enno Swart, Melina Niering

**Affiliations:** Medizinische Fakultät, Institut für Sozialmedizin und Gesundheitssystemforschung Magdeburg (Sachsen-Anhalt), Otto-von-Guericke-Universität Magdeburg, Leipziger Str. 44, 39120 Magdeburg, Deutschland

**Keywords:** Öffentlicher Gesundheitsdienst, Wissenschaft-Praxis-Kooperation, Gesundheitsberichterstattung, Prävention, Transferformate, Public health service, Science-practice cooperation, Health reporting, Prevention, Transfer formats

## Abstract

Vor dem Hintergrund des „Paktes für den Öffentlichen Gesundheitsdienst (Pakt ÖGD)“ prägt der Anspruch einer evidenzbasierten Arbeitsweise zunehmend die strategische Weiterentwicklung des ÖGD. Insbesondere auf kommunaler und Landesebene ist eine engere Zusammenarbeit zwischen Wissenschaft und Praxis erforderlich. In Sachsen-Anhalt wurde 2024 ein Kooperationsabkommen zwischen der Stadt Magdeburg und der Universitätsmedizin Magdeburg (UMMD) geschlossen, um die bestehende Zusammenarbeit strukturell zu verankern. Im selben Jahr folgte eine Projektvereinbarung mit weiteren ÖGD-Akteuren zur Etablierung einer nachhaltigen Struktur für den systematischen Austausch zwischen Wissenschaft und öffentlicher Gesundheitsverwaltung.

Dieser Artikel berichtet über die laufenden Kooperationen in Sachsen-Anhalt, die sich mit der Auswertung von Schuleingangsuntersuchungen, Detailanalysen der Frühen Hilfen, E‑Mental-Health-Interventionen für Kinder und Jugendliche sowie der Mpox-Bewältigung befassen. Sie verdeutlichen, wie eine regionale Zusammenarbeit konkret umgesetzt werden kann und welche Perspektiven sich für die Weiterentwicklung des ÖGD ergeben. Daneben spielen Transferformate wie regelmäßige Kooperationstreffen, wissenschaftliche Veranstaltungen und praxisorientierte Fortbildungen eine zentrale Rolle, um wissenschaftliche Erkenntnisse in die Praxis zu übertragen.

Die Erfahrungen aus Sachsen-Anhalt entsprechen den Empfehlungen des Beirats Pakt ÖGD, der u. a. Lehr- und Forschungsgesundheitsämter, Brückenprofessuren und nachhaltige Förderstrukturen fordert. Die Kooperationsbeispiele zeigen, dass eine zukunftsfähige Weiterentwicklung des ÖGD lokale Innovationen sowie überregionale Strukturen erfordert, die Wissenschaft und Praxis systematisch verbinden.

## Abstract (en)

Against the backdrop of the Pact for the Public Health Service (Pakt für den Öffentlichen Gesundheitsdienst; Pakt ÖGD), the commitment to evidence-informed practice is increasingly shaping the strategic development of Germany’s Public Health Service (ÖGD). Particularly at the municipal and federal state levels, closer collaboration between research and public health practice is needed. In Saxony-Anhalt, a cooperation agreement was concluded in 2024 between the City of Magdeburg and Magdeburg University Medicine (UMMD) to institutionally strengthen their existing collaboration. In the same year, a project agreement involving additional public health stakeholders was established to create a sustainable framework for systematic exchange between academia and public health administration.

This article presents ongoing collaborative initiatives in Saxony-Anhalt, including projects on the analysis of school entry examinations, in-depth evaluations of Early Childhood Intervention Services (Frühe Hilfen), e‑mental health interventions for children and adolescents, and the public health response to Mpox. These examples illustrate how regional cooperation between academia and practice can be operationalized and highlight the opportunities such collaborations offer for the continued development of the Public Health Service. In addition, knowledge translation formats such as regular coordination meetings, scientific events, and practice-oriented training play a key role in facilitating the transfer of evidence into public health practice.

The experiences from Saxony-Anhalt are consistent with the recommendations of the Advisory Board of the Pakt ÖGD, which has called, among other measures, for the establishment of teaching and research public health offices, bridge professorships, and sustainable funding structures. The examples of collaboration demonstrate that the future-oriented development of the Public Health Service requires both local innovation and supra-regional structures that systematically connect science and practice.

## Einleitung

Der Öffentliche Gesundheitsdienst (ÖGD) befindet sich in einem tiefgreifenden Wandel [1]. Mit dem 2018 von der Gesundheitsministerkonferenz (GMK; [2]) verabschiedeten Leitbild für einen modernen Öffentlichen Gesundheitsdienst wurde die Notwendigkeit einer engeren Verzahnung von Wissenschaft und Praxis hervorgehoben. Der Anspruch einer evidenzbasierten Arbeitsweise prägt seither die strategische Weiterentwicklung des ÖGD auf kommunaler, Landes- und Bundesebene. Die Beziehung zwischen ÖGD und Wissenschaft war historisch über Jahrzehnte distanziert und hat erst in den letzten Jahren wieder eine systematische Annäherung erfahren [3]. In diesem Kontext ist auch die Etablierung neuer Professuren für den Öffentlichen Gesundheitsdienst, etwa in Dresden und Leipzig, zu sehen [4, 5]. Diese Entwicklung erhielt durch den Pakt für den ÖGD seit 2020 zusätzliche Dynamik [6]. Sie spiegelt sich in neu etablierten bundesweiten Strukturen wider, darunter wissenschaftliche Fachgesellschaften (DGÖG, DGÖGB)^1^, Forschungs- und Evidenznetzwerke wie ÖGD-FORTE, erste Lehr- und Forschungsgesundheitsämter sowie Empfehlungen des „Beirats zur Beratung zukunftsfähiger Strukturen im Öffentlichen Gesundheitsdienst in Umsetzung des Paktes für den Öffentlichen Gesundheitsdienst“ – kurz „Beirat Pakt ÖGD“ zur strukturellen Weiterentwicklung des ÖGD [3, 6–8].

Vor diesem Hintergrund wird zunehmend deutlich, dass Kooperationen zwischen Gesundheitsämtern, Landesbehörden des ÖGD und wissenschaftlichen Einrichtungen erforderlich sind, um aktuelle Herausforderungen des ÖGD durch evidenzbasierte und praxisnahe Strategien zu bewältigen und explizit das wissenschaftliche Potenzial und damit dessen wissenschaftliche Ausrichtung zu stärken. Auch das Land Sachsen-Anhalt hat dafür umfangreiche Fördermöglichkeiten eröffnet. Mit einem im Juli 2024 geschlossenen Kooperationsabkommen zwischen der Stadt Magdeburg und der Universitätsmedizin Magdeburg (UMMD) wurde die zuvor gewachsene Zusammenarbeit strukturell verankert und auf eine strategische Grundlage gemeinsamer Forschungs- und Entwicklungsaktivitäten im Bereich der kommunalen Gesundheitsförderung gestellt. Im Dezember 2024 konnte eine parallel ausgearbeitete Projektvereinbarung mit verschiedenen Akteuren des ÖGD in Sachsen-Anhalt unterschrieben werden, in der die Zuständigkeiten, Aufgaben und Kommunikationsstrukturen verbindlich geregelt wurden. Ziel ist es, über die Projektförderung des Landes Sachsen-Anhalt hinaus eine dauerhafte Struktur für den systematischen Austausch zwischen Wissenschaft und öffentlicher Gesundheitsverwaltung zu schaffen.

Die vertraglich geregelte Zusammenarbeit entwickelte sich aus bestehenden Kontakten und gemeinsamen Aktivitäten des Instituts für Sozialmedizin und Gesundheitssystemforschung (ISMG), Medizinische Fakultät der Otto-von-Guericke-Universität Magdeburg, mit seinen Partnern im ÖGD. Die Kontakte entstanden ursprünglich im Kontext der akademischen Lehre und wurden auf vertraglicher Grundlage inhaltlich erweitert und strukturell gefestigt.

Dieser Artikel stellt die verschiedenen Kooperationen des ISMG und seiner Praxispartner vor, die exemplarisch zeigen, wie regionale Zusammenarbeit zwischen Wissenschaft und Praxis konkret umgesetzt wird und welche Ergebnisse sie hervorbringen kann. Zudem werden Transferaktivitäten dargestellt, die im Rahmen der gemeinsamen Arbeit seit 2024 entstanden sind. Im Anschluss wird die bisherige Zusammenarbeit reflektiert und Faktoren für das Gelingen einer langfristigen Kooperation werden diskutiert. Ziel ist es, am Beispiel Sachsen-Anhalts aufzuzeigen, wie wissenschaftliche und praktische Perspektiven im ÖGD sowie in der sozialmedizinischen und Versorgungsforschung synergistisch verknüpft werden können und zur wissenschaftlichen Profilierung des ÖGD beitragen.

## Bandbreite der Kooperation zwischen Wissenschaft und ÖGD in Sachsen-Anhalt

### Schuleingangsuntersuchungen

Im Rahmen der Schuleingangsuntersuchung (SEU) finden in Deutschland jährlich verpflichtende Vollerhebungen zur Gesundheit und Entwicklung einzuschulender Kinder statt. Während der COVID-19-Pandemie beeinflussten Eindämmungsmaßnahmen den sozialen Alltag von Kindern. Ebenso stellt die Integration von Kindern im Zuge der Fluchtmigration seit 2015 sowie infolge des Ukrainekriegs ab 2022 die Kommunen vor große Herausforderungen. Im Rahmen zweier Dissertationsvorhaben am ISMG werden mögliche Auswirkungen der COVID-19-Pandemie und der Fluchtmigration auf die Entwicklung und Gesundheit von Kindern näher untersucht. Der Anstoß dieses Projekts ging von einzelnen Gesundheitsämtern aus, die eine vertiefende Auswertung der SEU-Daten über die Gesundheitsberichterstattung des Landes hinaus als sinnvoll erachteten, um vor dem Hintergrund der beschriebenen Rahmenbedingungen Ansatzpunkte für zielgruppen- und stadteil- bzw. gemeindebezogene Fördermaßnahmen zu entwickeln.

Erste Ergebnisse zeigen im betrachteten Zeitraum unterschiedliche Trends in der Entwicklung von Kindern in den 5 Entwicklungsbereichen (Grobmotorik, Feinmotorik, Artikulation, Grammatik und geistige Entwicklung). In den sprachlichen Bereichen Artikulation und Grammatik sind seit 2018 deutliche Einbrüche zu verzeichnen. Zudem bestehen ausgeprägte Zusammenhänge zwischen dem Entwicklungszustand bzw. den festgestellten Förderbedarfen nach sozialem Status und Migrationshintergrund mit deutlich überdurchschnittlichem Förderbedarf bei Kindern aus Familien mit niedrigem sozialen Status und mit beidseitigem Migrationshintergrund.

Unsere Auswertungen weisen darauf hin, dass Alltageinschränkungen während der COVID-19-Pandemie die kindliche Gesundheit und Entwicklung beeinflusst haben könnten, insbesondere die sprachliche Kompetenz. Außerdem zeigt sich die Bedeutung der Identifizierung spezifischer Förderbedarfe, vor allem im sprachlichen Bereich, bei vulnerablen Gruppen wie Kindern mit Migrationshintergrund, wie auch empirische Studien andernorts belegen [9, 10]. Differenzierte Analysen der Daten der Schuleingangsuntersuchungen liefern für die regionalen gesundheitspolitischen Akteure und Institutionen zielgruppenorientierte Empfehlungen für Förderangebote im frühkindlichen Bereich, besonders in Bezug auf die sprachliche Entwicklung als wesentliche Determinante der sozialen Integration.

### Frühe Hilfen

In einem Teilprojekt wird die Inanspruchnahme der Frühen Hilfen im städtischen und ländlichen Kontext in Sachsen-Anhalt analysiert. Die Motivation der Initiatoren war, über die Standarddokumentation der Leistungen hinaus im wissenschaftlichen Austausch mit dem Partner ISMG eine fundierte Evaluation der eigenen Tätigkeit anzustoßen. Die Standarddokumentation der Frühen Hilfen erfasst sowohl die soziodemografische Struktur der Familien als auch die Gründe für Unterstützungsbedarfe. Die Frühen Hilfen sind ein deutschlandweit etabliertes Netzwerk, das werdenden/jungen Familien einen freiwilligen, niedrigschwelligen Zugang zu Unterstützungsangeboten bieten soll [11–13]. Sie sind regelmäßig Gegenstand der Forschung; es konnte gezeigt werden, dass sie wirksam sind, ihr volles Potenzial jedoch nicht ausschöpfen und viele Familien nicht erreichen [13].

In der Regel werden in Sachsen-Anhalt die Angebote der Frühen Hilfen durch das zuständige Jugendamt koordiniert [12]. In den vergangenen Jahren wurden umfangreiche Daten erhoben, die jedoch – anders als in anderen Städten – bislang nicht wissenschaftlich ausgewertet wurden. Ziel dieses Schrittes sind die Analyse häufiger Beratungsanlässe, ihrer Verläufe sowie eine Bewertung der erzielten Ergebnisse, um daraus im kontinuierlichen wissenschaftlichen Austausch zwischen ISMG und Jugendämtern Vorschläge zur Weiterentwicklung der Unterstützungsangebote abzuleiten. Hierfür wurden von 2 Jugendämtern Daten zu 330 abgeschlossenen Betreuungsfällen aus den Jahren 2020 bis 2024 in pseudonymisierter Form bereitgestellt. Diese wurden hinsichtlich soziodemografischer Merkmale, Zugangswege, Art und Intensität der Betreuung, Problemlagen sowie Umstände der Fallbeendigung beschrieben und im Hinblick auf den Einfluss von Belastungsfaktoren (z. B. Alleinerziehung, junge Mütter) untersucht.

Die meisten Fälle gehen auf Selbstmeldungen der Familien zurück, gefolgt von Zuweisungen durch Familienhebammen und Jugendämter. Über Kliniken und Kinderärzte werden vergleichsweise wenige Betreuungsfälle initiiert. Etwa die Hälfte der überwiegend weiblichen Hilfesuchenden ist jünger als 25 Jahre. Im Vergleich zur deutschen Gesamtbevölkerung sind Familien mit 3 oder mehr Kindern überrepräsentiert. Bildungsferne und sozioökonomisch schwache Bevölkerungsgruppen machen den Hauptteil der Betreuungsfälle aus. In nahezu allen Familien werden Erziehungsschwierigkeiten berichtet, gefolgt von ungünstigen sozialen Lebensbedingungen sowie psychischen Erkrankungen und Suchtproblematiken der Eltern.

Zwei Drittel der Fallbeendigungen erfolgen regelhaft; die Fachkräfte bewerten die Situation dabei überwiegend als verbessert, vor allem in Familien mit Migrationshintergrund. Weiterführende Hilfen werden häufig bei Suchtproblematiken, allein oder in Kombination mit psychischen Erkrankungen, vermittelt. Eine Verbesserungsquote von über 60 % spricht für die Wirksamkeit der Frühen Hilfen. Es ist davon auszugehen, dass dadurch in vielen Fällen ein intensiveres Eingreifen der Jugendämter vermieden werden kann. Das Potenzial der Frühen Hilfen ist jedoch noch nicht ausgeschöpft. Ein wichtiger Ansatzpunkt ist die stärkere Einbindung von Ärztinnen und Ärzten, die Unterstützungsbedarfe für werdende Mütter sowie Familien mit Kleinkindern frühzeitig erkennen können.

### E-Mental-Health-Interventionen für Kinder und Jugendliche

Vor dem Hintergrund des zunehmenden Bedarfs an psychotherapeutischer Versorgung und des bislang begrenzten Angebots zertifizierter, evidenzbasierter digitaler Interventionen für Kinder und Jugendliche [14–17] untersucht eine qualitative Studie Chancen, Herausforderungen und Gestaltungsmöglichkeiten von E‑Mental-Health-Interventionen (internet- und mobilbasierte Interventionen, z. B. Digitale Gesundheitsanwendungen (DiGA), Teletherapie) in dieser Zielgruppe. Darüber hinaus zielt die Studie darauf ab, die aktuelle psychische Gesundheit von Kindern und Jugendlichen zu analysieren, die Bedeutung frühzeitiger Prävention und Gesundheitsförderung zu unterstreichen und die Relevanz des übergeordneten Forschungs- und Handlungsfeldes Public Mental Health in Sachsen-Anhalt zu verdeutlichen. In die Analyse wurden neben Expert:innen aus der psychotherapeutischen Versorgung auch Perspektiven aus dem ÖGD, unter anderem eine Fachkraft für Psychiatriekoordination, sowie wissenschaftliche Mitarbeiter:innen einbezogen (*N* = 7).

Die befragten Expert:innen weisen auf die Notwendigkeit einer Stärkung des Stellenwerts von Public Mental Health in Sachsen-Anhalt hin. E‑Mental-Health-Angebote werden als potenzielle Entlastung der Versorgung bewertet, insbesondere durch ihre Flexibilität, Niedrigschwelligkeit und alltagsnahe Anwendung. Gleichzeitig bestehen zentrale Herausforderungen in Bezug auf technische und finanzielle Rahmenbedingungen, Datenschutz, Akzeptanz und altersgerechte Gestaltung. Dabei zeigen sich Unterschiede zwischen Akteur:innen aus dem ÖGD und anderen Versorgungskontexten in der Einschätzung der Umsetzbarkeit und der Implementierungsbedingungen von E‑Mental-Health-Interventionen. Zudem wird deutlich, dass der Einsatz digitaler Interventionen frühestens ab einem Alter von 14 Jahren als sinnvoll erachtet wird und die Anwendung individuell angepasst sowie im Sinne einer „Blended Therapy“ oder eines „Stepped-Care-Modells“ [17] erfolgen sollte. In der psychotherapeutischen Praxis können E‑Mental-Health-Angebote als ergänzende Instrumente in Prävention, Behandlung und Rezidivprophylaxe eingesetzt werden; sie ersetzen jedoch nicht den direkten therapeutischen Kontakt, der insbesondere im Kindes- und Jugendalter von hoher Bedeutung ist.

Der ÖGD nimmt in diesem Zusammenhang eine zentrale Schnittstellenfunktion ein. Beispielsweise bietet er über die kinder- und jugendpsychiatrischen und sozialpsychiatrischen Dienste niedrigschwellige Beratung, Kriseninterventionen und Weitervermittlungen an und leistet damit einen wichtigen Beitrag zur frühzeitigen Erkennung psychischer Belastungen [18]. Aus Sicht der im ÖGD tätigen Expert:innen wird das Potenzial insbesondere in der Niedrigschwelligkeit des Angebots gesehen. Darüber hinaus werden digitale Interventionen als mögliche Ergänzung zu bisherigen Versorgungszugängen insbesondere im ländlichen Raum gesehen. Perspektivisch könnte der ÖGD verstärkt in die Vermittlung, Erprobung und Aufklärung über E‑Mental-Health-Interventionen eingebunden werden, etwa durch Präventionsmaßnahmen und Informationsarbeit in Schulen, Kindertageseinrichtungen und kommunalen Settings. Auf diese Weise kann frühzeitig einer Stigmatisierung psychischer Erkrankungen im Sinne von Public Mental Health entgegengewirkt werden. Mit Blick auf den wachsenden Versorgungsbedarf ist es erforderlich, altersgerechte, störungsspezifische und wissenschaftlich fundierte digitale Interventionen systematisch in bestehende Versorgungsstrukturen zu integrieren. Eine enge Verzahnung zwischen Forschung, psychotherapeutischer Versorgung und dem ÖGD erscheint hierbei als wesentliche Voraussetzung für eine nachhaltige Implementierung. Eine zentrale Herausforderung bleibt dabei die direkte Erreichbarkeit minderjähriger Personen, insbesondere außerhalb institutioneller Kontexte.

### Mpox-Bewältigung im ÖGD

In einer Mixed-Methods-Studie mit qualitativen Interviews und einer quantitativen Befragung von Gesundheitsämtern wird die Rolle des ÖGD in Sachsen-Anhalt und Deutschland während des Mpox-Ausbruchs ab dem Jahr 2022 untersucht. ÖGD-Mitarbeitende wurden aktiv in die theoriegeleitete Entwicklung, Validierung und Weiterentwicklung der Erhebungsinstrumente sowie in die Interpretation der Ergebnisse eingebunden. Ergänzend wurde ein Scoping-Review durchgeführt, der nationale Erkenntnisse zur Mpox-Bewältigung systematisch zusammenfasst. Ziel ist es, den Umgang des ÖGD mit dem Mpox-Ausbruch zu erfassen, zentrale Herausforderungen, Strategien und Unterstützungsbedarfe zu identifizieren und daraus übertragbare Erkenntnisse für die Weiterentwicklung des Public-Health-Managements in Deutschland abzuleiten.

Die qualitativen Interviews (*N* = 7) zeigten, dass die Gesundheitsämter in Sachsen-Anhalt trotz paralleler Belastungen durch die COVID-19-Pandemie beim Mpox-Ausbruch engagiert und lösungsorientiert agierten. Besonders betont wurden Herausforderungen in der Impfstoffkoordination, der Kommunikation mit Betroffenen sowie der Kontaktpersonennachverfolgung. Es wurde hervorgehoben, dass die fehlende Verfügbarkeit von Isolationsmöglichkeiten und die Zurückhaltung ärztlicher Praxen innerhalb der medizinischen Versorgung zusätzliche Hürden darstellten. In ländlichen Regionen wurde zudem auf die eingeschränkte Anonymität hingewiesen. Die Ergebnisse wurden im Rahmen einer Expert:innenvalidierung gemeinsam mit den Befragten reflektiert und kontextualisiert. Zugleich zeigte sich, dass etablierte Netzwerke, kollegiale Unterstützung und Erfahrungen aus der COVID-19-Pandemie zur erfolgreichen Bewältigung beitrugen.

Die Ergebnisse der deutschlandweiten Befragung von Mitarbeitenden aus Gesundheitsämtern (*N* = 104) zeigen im Rahmen bivariater inferenzstatistischer Analysen signifikante Zusammenhänge zwischen potenziellen Einflussfaktoren und dem Sicherheitsempfinden bei der Mpox-Bewältigung. Kenntnisse über die Epidemiologie von Mpox waren dabei hochsignifikant mit einem höheren Sicherheitsempfinden assoziiert (*p* < 0,001). Befragte, die die interne Zusammenarbeit während des Mpox-Ausbruchs als sehr gut oder gut bewerteten, gaben signifikant häufiger an, sich sicher zu fühlen, als jene mit neutraler oder negativer Einschätzung (*p* = 0,004). Gleiches galt für die wahrgenommene kollegiale Unterstützung (*p* = 0,006) sowie für die externe Kooperation mit anderen Behörden oder Institutionen (*p* = 0,013).

Der Scoping-Review, basierend auf 3323 identifizierten Publikationen (01/2022–11/2025) und 4 eingeschlossenen Arbeiten, zeigt, dass Gesundheitsämter in Deutschland auf pandemieerprobte Strukturen und digitale Systeme zurückgreifen konnten, zugleich aber mit Herausforderungen wie Diagnoseunsicherheiten und umfangreicher Kontaktpersonennachverfolgung konfrontiert waren [19, 20]. Rein symptomorientierte Kontrollstrategien besitzen vermutlich nur eine begrenzte Wirksamkeit, da viele Infektionen unerkannt und undiagnostiziert bleiben [21]. Niedrigschwellige Impfangebote [21], koordinierte Kommunikation [19] und eine starke sektorübergreifende Zusammenarbeit im Sinne des One-Health-Ansatzes werden als zentrale Strategien hervorgehoben [22].

### Transferformate zwischen Wissenschaft und Praxis

Neben den Forschungsprojekten spielen Veranstaltungs- und Dialogformate eine wichtige Rolle, um die Zusammenarbeit zwischen Wissenschaft und ÖGD zu vertiefen. Vor diesem Hintergrund finden monatliche Kooperationstreffen mit den Vertragspartnern statt, die eine kontinuierliche Abstimmung und Bedarfsidentifikation ermöglichen.

Darüber hinaus stärken Austausch- und Netzwerkformate den Wissenstransfer. Mit der Reihe „ÖGD trifft Wissenschaft“ wurde zudem ein institutionelles Forum etabliert, das aktuelle Forschungsprojekte sichtbar macht und evidenzbasierte Ansätze in die Praxis trägt.

Auch Fortbildungen für Mitarbeiter:innen des ÖGD in Sachsen-Anhalt bieten Gelegenheit, Forschungsergebnisse praxisnah zu diskutieren und Bedarfe aus den Gesundheitsämtern zu thematisieren. Dadurch entsteht ein bidirektionaler Wissenstransfer, der eine stärker bedarfsorientierte Gesundheitsberichterstattung unterstützt.

Die wissenschaftlichen Ergebnisse wurden zudem auf nationalen Fachkongressen vorgestellt (ÖGD-Kongress 2025, Jahrestagung der Deutschen Gesellschaft für Sozialmedizin und Prävention – DGSMP 2025). Thematisch reichten die Beiträge von Datenanalysen zu Schuleingangsuntersuchungen über E‑Mental-Health-Interventionen bis hin zur Bewältigung von Mpox im ÖGD [23–27]. Damit wurde die Bandbreite der Kooperation zwischen Wissenschaft und Praxis auch überregional sichtbar.

Besondere Sichtbarkeit erlangten die geschilderten Einzelprojekte durch die gemeinsame Ausgestaltung eines Workshops mit dem Bundesinstitut für Öffentliche Gesundheit (BIÖG) im Rahmen des 3. Symposiums des BIÖG unter dem Titel „Verstehen – kooperieren – beteiligen: Partizipation als Schlüssel einer innovativen Prävention und Gesundheitsförderung“ am 18.09.2025 in Berlin. Der Beitrag des ISMG diente der Vertiefung der Diskussion über partizipative Ansätze in der Prävention und Gesundheitsförderung und verdeutlicht die bundesweite Anerkennung der Kooperation zwischen Wissenschaft und ÖGD als praxisnahes Modell zur Förderung evidenzbasierter und partizipativer Public-Health-Strukturen.

## Reflexion und Bewertung der bisherigen Zusammenarbeit

Mit den hier präsentierten Ergebnissen aus der 2‑jährigen Zusammenarbeit der Projektpartner erscheint der Anspruch einer engeren Kooperation zwischen Wissenschaft und Praxis bereits praktisch umgesetzt. Das ISMG arbeitet in enger Abstimmung mit den Projektpartnern daran, Bedarfe an wissenschaftlich fundierten Informationen zu identifizieren, evidenzbasierte Erkenntnisse in der Praxis anzuwenden und planungsrelevante Daten systematisch zu erheben. Modellprojekte werden gemeinsam entwickelt und durchgeführt. Dadurch entsteht zudem die Perspektive einer langfristigen Verstetigung. Die Erfahrungen aus Sachsen-Anhalt stehen im Einklang mit den Empfehlungen des Beirats Pakt ÖGD, der eine nachhaltige Verzahnung von Wissenschaft und Praxis über verschiedene Disziplinen hinweg fordert [1].

Darüber hinaus werden die Ergebnisse und Erfahrungen durch gemeinsame Veranstaltungen in die akademische Lehre eingebunden, um Studierende frühzeitig mit den Anforderungen, Chancen und Herausforderungen des ÖGD vertraut zu machen. So vermitteln Vertreter:innen der Projektpartner u. a. in Seminaren des Faches Sozialmedizin im klinischen Studienabschnitt Aufgaben, Inhalte, Herausforderungen und Lösungen sowie methodische Herangehensweisen in ihrer täglichen Arbeit und gehen damit weiter als die meisten medizinischen Fakultäten in Deutschland [28]. Eine stärkere Berücksichtigung des ÖGD in Lehrveranstaltungen des Humanmedizinstudiums wäre wünschenswert, bleibt jedoch künftigen Änderungen der Approbationsordnung vorbehalten.

Die bisherigen Erfahrungen in Sachsen-Anhalt verdeutlichen, wie wissenschaftliches Arbeiten mit den Aufgaben des ÖGD kombiniert werden kann. Zugleich zeigt sich ein weiterer Bedarf an wissenschaftlicher Unterstützung. Die Gesundheitsämter in Sachsen-Anhalt wenden sich zunehmend mit spezifischen Fragestellungen an die wissenschaftlichen Kooperationspartner. Diese Entwicklung verweist einerseits auf ein wachsendes Interesse an evidenzbasierten Ansätzen, verdeutlicht andererseits aber auch die begrenzten Ressourcen vieler Ämter, die stark durch gesetzliche Pflichtaufgaben gebunden sind. Perspektivisch besteht Bedarf, die Kompetenz im ÖGD gezielt und anhaltend wissenschaftlich zu verbessern.

## Förderung einer nachhaltigen Zusammenarbeit

Der Beirat Pakt ÖGD benennt verschiedene Förderformate zur Unterstützung des ÖGD, darunter projektbezogene Ausschreibungen im Rahmen klassischer Forschungsförderung, Professuren und Brückenprofessuren für Öffentliches Gesundheitswesen (ÖGW), bereichsübergreifende Rotationsstellen sowie geteilte Stellenmodelle zwischen Hochschulen und Gesundheitsämtern [6]. Eine dauerhaft gesicherte Finanzierung dieser Strukturen wird als unabdingbar angesehen.

Ein zentrales Element der Empfehlungen des Beirats Pakt ÖGD bilden Lehr- und Forschungsgesundheitsämter [29]. Sie sollen als innovative Schnittstelle zwischen Praxis, universitärer Lehre und Forschung wissenschaftlich qualifiziertes Personal einbinden und kleinere Gesundheitsämter fachlich unterstützen. Empfohlen wird, einen Teil der neu zu schaffenden wissenschaftlichen Stellen in Kooperation mit Hochschulen dort anzusiedeln, um praktische und wissenschaftliche Tätigkeiten im ÖGD sowie in Forschung und Lehre zu verbinden. Die in diesem Artikel beschriebene vertragliche Kooperation des ÖGD mit einer medizinischen Fakultät kann als Ausgangspunkt für eine beispielhafte, langfristig angelegte Entwicklung in Sachsen-Anhalt dienen. Ziel ist die nachhaltige Stärkung größerer wissenschaftlich orientierter Gesundheitsämter und ihrer regionalen Netzwerke. In der Konsequenz sollten wissenschaftliche erfahrene Mitarbeiter:innen des ÖGD verstärkt Leitungsfunktionen mit inhaltlichem Gestaltungsspielraum übernehmen.

Die Auswertung der laufenden Projekte zeigt in methodischer Hinsicht am Beispiel der Daten der Schuleingangsuntersuchungen, dass insbesondere kleinräumige Analysen für die Arbeit des ÖGD von zentraler Bedeutung sind. Durch die Erfassung und Aufbereitung kleinräumiger Datensätze können lokale Unterschiede in der gesundheitlichen Lage sichtbar gemacht werden, etwa in Bezug auf soziale Determinanten, Inanspruchnahme von Präventionsangeboten oder Belastungsfaktoren in bestimmten Stadtteilen. Diese Daten, die den Gesundheitsämtern in Sachsen-Anhalt in den Kommunal- und Grundschulreports vom LAV (Landesamt für Verbraucherschutz Sachsen-Anhalt) zur Verfügung gestellt werden, ermöglichen es, gesundheitliche Bedarfe differenziert zu beschreiben und die Grundlage für gezielte, standortbezogene Maßnahmen zu schaffen. Hier können wissenschaftliche Expertise und Beratung dazu beitragen, den Fokus der Gesundheitsberichterstattung des Landes zu erweitern, um Gründe für spezifische Entwicklungen in definierten vulnerablen Gruppen zu untersuchen, Lösungsansätze zu erforschen und Akteure darin zu unterstützen, diese Erkenntnisse in zielgruppenspezifischen Handlungsempfehlungen zu kommunizieren.

Des Weiteren erfordert die nachhaltige Zusammenarbeit von Wissenschaft und Praxis hochqualifiziertes Personal. Neben strukturierten Master‑, Promotions- und Habilitationsprogrammen sind daher spezifische postgraduelle Qualifizierungsangebote notwendig. Die vom Bundesministerium für Gesundheit geförderten Projekte geben bereits wichtige Impulse zur inhaltlichen Ausgestaltung solcher Programme [7].

Zukünftig sollten die im Rahmen des „Paktes ÖGD“ formulierten Zielsetzungen einer intersektoralen und interdisziplinären Zusammenarbeit weitergeführt werden [8]. Dabei kann aus den Erfahrungen in Sachsen-Anhalt gelernt werden, wie wissenschaftliche Strukturen systematisch in die Tätigkeiten des ÖGD integriert werden können. Auf Bundes- und Landesebene braucht es jedoch klare strukturelle und finanzielle Voraussetzungen, damit diese Ansätze verstetigt und verbreitet werden können. Eine Verstetigung des Paktes für den ÖGD über das Jahr 2026 hinaus sowie der gezielte Ausbau von Lehr- und Forschungsgesundheitsämtern, Brückenprofessuren und Förderprogrammen stellen zentrale Bausteine dieser Entwicklung dar. Die Verzahnung von Praxis und Wissenschaft ist eine notwendige Voraussetzung, um den ÖGD zukunftsfähig zu gestalten und seine Rolle im Gesundheitssystem zu stärken. Die im Bericht des Beirats Pakt ÖGD (2023) formulierten Empfehlungen unterstreichen dabei die Bedeutung interdisziplinärer Forschungs- und Kooperationsstrukturen [6]. Der beschriebene Ansatz greift diese Zielrichtung auf, indem er unterschiedliche Fachperspektiven in gemeinsame Projekte integriert und somit exemplarisch zeigt, wie interprofessionelle Zusammenarbeit im ÖGD praktisch realisiert werden kann.

Auch wenn sich diese Strukturen bundesweit noch im Aufbau befinden, zeigen die Erfahrungen aus Sachsen-Anhalt, dass die institutionelle Verankerung gemeinsamer Projekte zwischen Universität, Gesundheitsämtern, anderen Institutionen der Kinder- und Jugendhilfe und Landesbehörden des ÖGD einen entscheidenden Schritt in diese Richtung darstellt. Die grundlegenden Rahmenbedingungen wurden geschaffen; diese Strukturen sollten nun gezielt weiterentwickelt und langfristig verstetigt werden.

## Fazit

Das Beispiel Sachsen-Anhalt zeigt, wie Kooperationen zwischen Praxis und Wissenschaft im ÖGD erfolgreich umgesetzt werden können. Der aktuelle Fokus liegt auf kleinräumigen Datenanalysen, evidenzbasierten Ansätzen und partizipativen Projekten. Mittelfristig sollte über den Pakt hinaus das wissenschaftliche Potenzial gestärkt und die Zusammenarbeit mit der Wissenschaft verstetigt werden.

Diese Projekte verdeutlichen die Bandbreite der wissenschaftlichen Arbeit in Sachsen-Anhalt. Gemeinsam ist den Ansätzen, dass sie Datenanalysen im Verbund, evidenzbasierte Erkenntnisse und interdisziplinäre Kooperationen für die Weiterentwicklung des ÖGD nutzbar machen.

Die Empfehlungen des Beirats Pakt ÖGD geben den strategischen Rahmen, um solche Modelle bundesweit zu etablieren. Ein zukunftsfähiger ÖGD erfordert lokale Innovation ebenso wie überregionale Strukturen, die Wissenschaft und Praxis systematisch miteinander verbinden.

## Data Availability

Die Daten, die analysiert wurden, sind auf Anfrage beim entsprechenden Autor erhältlich.

## Footnotes

1 DGÖG: Deutsche Gesellschaft für Öffentliches Gesundheitswesen e. V., DGÖGB: Deutsche Gesellschaft für Öffentliche Gesundheit und Bevölkerungsmedizin e. V.
